# Supplementary material for: An updated proteomic analysis of Drosophila haemolymph after bacterial infection
Source: Fly (Austin). 2025 Apr 13;19(1):2485685. doi: 10.1080/19336934.2025.2485685 (PMC12005426; doi:10.1080/19336934.2025.2485685)
Supplement: Supplemental Material [file KFLY_A_2485685_SM7039.docx]

**Supplementary Figure 1**

**Figure S1:** (A) Venn diagram showing the overlap of protein groups with decreased abundance after infection with *Ecc15* (left) or *M. luteus* (right). (B,C) Comparison of changes in protein abundance in the hemolymph 24 hours after *Ecc15* (B) or *M. luteus* (C) infection to the change in transcript levels 12 hours after the same infection. Data are plotted as log2 of the fold change (FC) of the infected over unchallenged condition. The most highly dysregulated genes/proteins are highlighted on the graph.

**
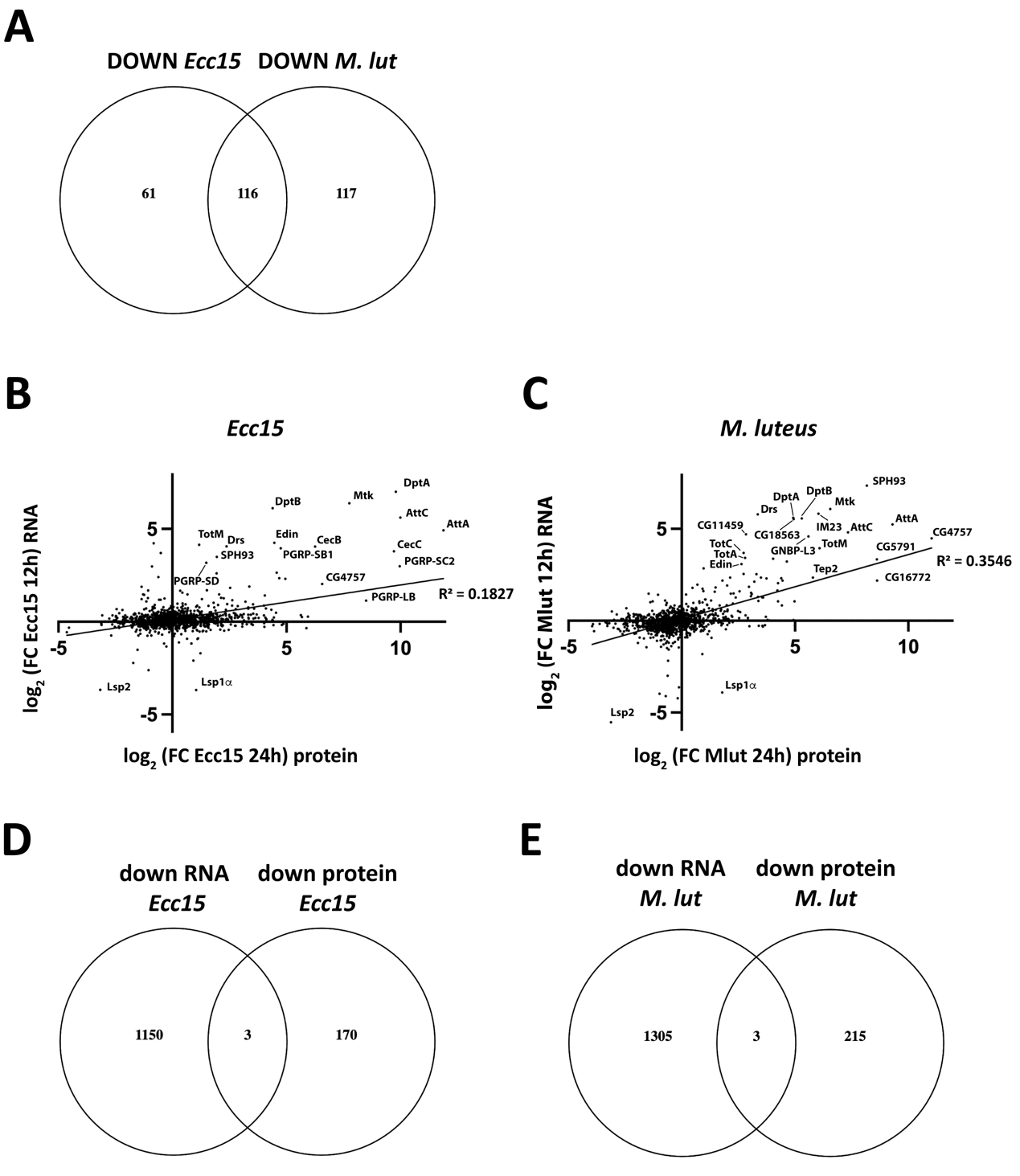
**
